# Lipid Metabolism in Late-Onset Alzheimer’s Disease Differs from Patients Presenting with Other Dementia Phenotypes

**DOI:** 10.3390/ijerph16111995

**Published:** 2019-06-05

**Authors:** Syena Sarrafpour, Cora Ormseth, Abby Chiang, Xianghong Arakaki, Michael Harrington, Alfred Fonteh

**Affiliations:** 1 Huntington Medical Research Institutes, Pasadena, CA 91105, USA; syena.sarrafpour@gmail.com (S.S.); cora.ormseth@gmail.com (C.O.); jchiang@coh.org (A.C.); xianghong.arakaki@hmri.org (X.A.); michael.harrington@hmri.org (M.H.); 2 School of Medicine, Tufts University, Medford, MA 02155, USA; 3 Department of Neurology, Yale University, New Haven, CT 06520, USA; 4 Beckman Research Institute, City of Hope, Duarte, CA 91010, USA

**Keywords:** amyloid, ceramide, cerebrospinal fluid, late-onset Alzheimer’s disease, lysophosphatidylcholine, other dementia, phosphatidylserine, phospholipase A_2_, sphingomyelinase, Tau proteins

## Abstract

Abnormal cerebrospinal fluid (CSF) levels of β-amyloid peptides (Aβ_42_) and Tau and cognitive decline are typical characteristics of Alzheimer’s disease (AD). Since dysregulation in lipid metabolism accompanies abnormal amyloid formation, we quantified glycerophospholipids (GP) and sphingolipids (SP) in CSF fractions from participants with late-onset AD (LOAD, *n* = 29) or with Other Dementia (OD, *n* = 10) to determine if alterations in lipid metabolism account for pathological differences. Aβ_42_ and total Tau levels were determined using a sandwich ELISA. Liposomal-based fluorescent assays were used to measure phospholipase A_2_ (PLA_2_) and acid or neutral sphingomyelinase (aSMase, nSMase) activities. Supernatant fluid (SF) and nanoparticle (NP) lipids were quantified using LC-MS/MS. Although CSF Aβ_42_ and Tau levels are similar, phosphatidylserine (PS) in SF and ceramide (CM) levels in NP are significantly higher in OD compared with LOAD. The aSMase but not the nSMase activity is higher in OD. PLA_2_ activity in CSF from OD subjects positively correlates with several GP classes in SF and NP fractions but not in LOAD fractions. Our data indicate differences in CSF lipid metabolism between dementia variants. Higher levels of inflammatory and apoptotic lipids may induce faster neuronal death, resulting in the earlier cognitive decline in patients with OD phenotypes.

## 1. Introduction

Late-onset Alzheimer’s disease (LOAD) is the most common neurodegenerative disorder characterized by progressive loss of cognitive function that interferes with daily activities [1]. The pathology involves neurofibrillary tangles, composed of hyperphosphorylated Tau proteins and β-amyloid (Aβ) plaques, which obstruct proper synapse function and lead to neuronal cell loss and atrophy [1]. The most significant risk factor is age: the risk doubles every five years after the age of 65, with a higher AD prevalence in females than males [2,3]. There is also a strong genetic component that indicates an increase in dementia risk in individuals expressing the E4 isoform of ApoE gene [4]. ApoE modulates Aβ metabolism and transport and thus prevents the formation of toxic Aβ fibrils [5]. Current biomarkers of AD include elevated CSF T-Tau, elevated P-Tau, and diminished Aβ_42_ levels [6,7,8]. However, these biomarkers are more readily used to distinguish AD patients from non-AD patients rather than differentiating between AD and other dementias.

In addition to LOAD, Frontotemporal Dementia (FTD), Dementia with Lewy Body (DLB), and vascular dementia (VaD) account for a substantial amount of cases of other dementias. FTD constitutes 5–15% of all dementia cases and is the second most common cause of dementia for patients under the age of 65 [9]. Unlike LOAD, FTD patients have relative preservation of memory; nonetheless, they experience progressive deterioration in language, behavior, and personality [9]. The genetic background of FTD lies on two mutations on the microtubules associated protein Tau gene (MAPT) and progranulin (GRN) gene, respectively [9]. DLB is yet another common type of dementia that is characterized by the presence of Lewy bodies in the brainstem, throughout the amygdala and the cortex [10]. Lewy bodies are composed of accumulated α-synuclein inside the nuclei of neurons. Symptoms are typically similar to that of AD, although DLB patients experience visual hallucinations, visuospatial deficits, fluctuating cognitive awareness, and parkinsonian symptoms [10,11,12]. Memory impairment is more pronounced in AD than DLB, whereas SPECT and PET scans have demonstrated hypometabolism and hypoperfusion in the primary visual cortex in DLB but not in AD [13]. 

Lipids are involved in critical neurobiological functions, such as membrane formation, cellular binding and recognition, transport, energy, and signaling [14,15]. In AD, lipid biochemistry is dysregulated, causing profound effects on signaling and Aβ plaque accumulation [16,17,18,19,20,21]. Glycerophospholipid (GP), a main component of membranes, are reduced in AD patients, which is attributable to abnormal phospholipid metabolism and turnover. More specifically, lipid characterizations have revealed decreased phosphatidylcholine (PC) and phosphatidylethanolamine (PE) in the frontal and parietal cortex of AD, while charged GPs on cellular membranes directly interact with and influence the release of Aβ peptides from cells [22]. In AD, changes in GPs in cerebrospinal fluid (CSF) accompany an increase in phospholipase activity [23]. There is significant stabilization of nonspecific β sheets through this interaction, and a thin polar dehydrated film is created by ganglioside GM1 to promote Aβ aggregation [24]. Sphingolipids (SPs) are essential for signal transmission through their roles as extracellular receptor ligands and intracellular secondary messengers [25,26]. SP metabolism is important in AD by disrupting protein-lipid interactions as well as membrane trafficking and signaling [27]. 

Several enzymes, as well as lipids, interact with Aβ metabolism [19,28,29,30,31,32]. Oxidative stress has been shown to stimulate sphingomyelinase (SMase), an enzyme that converts sphingomyelin (SM)to ceramide (CM) [33,34]. CM, a second messenger and regulator of apoptosis, stabilizes β secretase activity [31]. In AD, increased CM levels regulate β and γ secretases and amyloid precursor protein (APP) processing on lipid rafts [35]. Mislocalization of β and γ secretase to lipid rafts in post Golgi rather than lysosomes can promote Aβ accumulation [35]. Sphingosine is associated with apoptosis and is elevated in AD brains [36]. Sphingosine also plays a role in vesicle fusion and exocytosis [37]. Increased cholesterol increases the risk of AD, and more cholesterol in membranes has been associated with larger lipid raft size and accelerated α and β secretase activity [38,39,40].

The huge role of lipid metabolism in dementia and differences in clinical manifestation of diseases led us to hypothesize that lipid metabolism may differentiate dementias. This project aims to determine if lipid metabolism can differentiate LOAD from other dementias (OD), such as FTD and DLB. This characterization will allow for further research on early biomarkers of dementia subtypes. It would provide insight into any possible metabolic pathways, early diagnosis, and effective treatment of these diseases.

## 2. Materials and Methods

### 2.1. Study Participants

Human study participants were enlisted through newspaper advertising and visits to senior centers and assisted living residences. Criteria for inclusion required participants to be between the ages of 45 to 100 years old and diagnosed with LOAD [41], DLB, FTD, or vascular dementia (VaD). Criteria for exclusion comprised of patients taking anticoagulants or having contraindications against lumbar punctures. The local Internal Review Board of Huntington Memorial Hospital approved the protocol (FWA2338), and we obtained a signed informed consent form from each study participant. A Uniform Data Set was used to obtain all data sets within one month as described by the National Alzheimer’s Coordinating Center [42,43], and included structured clinical interviews; list of over the counter medications, nutritional supplements, and prescription drugs; physical examinations focusing on risk factors of dementia associated with the neurological and cardiovascular systems. We performed Clinical MRI on a 1.5T GE scanner to exclude significant vascular or neoplastic conditions. Clinical tests for study participants included the Mini-Mental State Exam [44], Montreal Cognitive Assessment [45], and Clinical Dementia Rating (CDR) [46]. A clinically probable diagnosis of the human participants was concluded after fulfillment of current criteria of LOAD [47] and other dementias based on consensus criteria of DLB [48] behavior variant FTD [49] and VaD [50]. The clinically probable dementia (LOAD, DLB, FTD, VaD, or mixed dementia) classification was established after the exam workup previously mentioned with triple scoring of neuropsychological test independently by research staff and clinical conferencing by at least three faculty clinicians [51]. 

### 2.2. ApoE Genotyping

ApoE genotyping was performed using a polymerase chain reaction mixture of primers specific for E2, E3, and E4, using the protocol described by Calero et al. [52]. We created a scale to quantify the participants’ risk from ApoE4: E2/E2 = 0, E2/E3 = 1, E3/E3 = 2, E2/E4 = 3, E3/E4 = 4, E4/E4 = 5. 

### 2.3. Quantification of CSF Total Protein, Tau, and Aβ_42_

A clinician obtained lumbar CSF between 8:00 am and 10:00 am after an overnight fast, and laboratory personnel removed cellular debris by centrifugation at 1000 g for 2 min. We measured CSF levels of Aβ_42_ and total Tau using a sandwich ELISA kit (Innotest β-amyloid_1-42_ and Innotest hTAU-Ag, Innogenetics, Gent, Belgium) as previously described [51]. We determined total protein concentration in CSF using a fluorescent protein assay kit (Quant^IT^, Invitrogen/Molecular Probes, Eugene, OR, USA) using serum albumin (0–500 ng/mL) as a standard as previously described [51].

### 2.4. Enzyme Activity Assays

To measure phospholipase A_2_ (PLA_2_), acid sphingomyelinase (aSMase), or neutral sphingomyelinase (nSMase) activity in the CSF samples, we used a modified liposomal-based fluorescent assay with 7-hydroxycoumarinyl-arachidonate (0.3 mM), 7-hydroxycoumarinyl-linolenate (0.3 mM), hydroxycoumarinyl-6-hyptenoate (0.3 mM), 10 mM dioleoylphophatidylcholine (DOPC), and dioleoylphosphatidylglycerol (DOPG) (10 mM) as substrates [23]. To initiate PLA_2_ activity, we added CSF containing 10 µg total protein to 96 well plates containing 50 µL PLA_2_ substrates and 50 mM Tris HCl buffer (pH 8.9). PLA_2_ activity was measured using an excitation wavelength of 360 nm and emission at 460 nm, and specific activity was calculated as the relative fluorescent units (RFU)/µg protein/min) for CSF samples. 

aSMase and nSMase activities in CSF were determined using an Amplex red fluorometric assay following the recommended protocol obtained from the kit’s manufacturer (Invitrogen/Molecular Probes, Eugene, OR, USA). Activities are presented as relative fluorescent units per minute for a 10 µg protein applied in duplicates on 96 well plates [53]. 

### 2.5. Lipid Extraction

We prepared lipid fractions consisting of supernatant fluid (SF) and nanoparticles (NP), according to Harrington et al. [54]. After the addition of internal standards and retention time calibrants (5 ng PC (11:0/11:0), 1 ng D_4_-PAF, 5 ng lysophosphatidylcholine (LPC) (11:0), and 5 ng D_4_-LPC) to 1 mL SF, lipids were extracted using a modified Bligh & Dyer protocol [55]. Briefly, we added 2 mL of methanol containing 0.2 mg/mL BHT and 1 mL of chloroform to 1 mL of CSF. After sonication of the resulting mixture for 10 min, we added 1 M NaCl (1 mL) to facilitate the separation of a lipid-rich chloroform layer. We performed a similar extraction procedure on the NP fraction after resuspension in 1 M NaCl solution. After drying under N_2_, we reconstituted lipid extracts in HPLC solvent before LC-MS/MS analyses.

### 2.6. LC-MS/MS of Lipids

We separated lipid extracts on a TSK-Gel Amide-80 Column (2.0 × 150 mm) using a binary solvent system as previously described [23,53]. After LC, lipids were positively ionized in electrospray ionization (ESI) and detected using several MS scanning modes in a triple quadrupole mass spectrometer (TSQ Classic, Thermo Fisher Scientific, San Jose, CA, USA) [23,53]. We optimized all GP and SP scans to their respective collision energies and collected data using three different scan windows from 0–6 min for CM, dihydroceramide (dhCM), PE, unknown lipid containing phosphoethanolamine (xPE), and N-acyl phosphatidylethanolamine (NAPE), 6–14.5 min for PC, SM, phosphatidylserine (PS), and platelet-activating factor (PAF), and 14.5–40 min for LPC. 

### 2.7. LC-MS/MS Data Analysis

We used the Qual Browser module of the Xcalibur software (Thermo Fisher, San Jose, CA, USA) for peak integration of GP and SP classes. All integrated peaks were normalized to the internal standard (IS), PC (11:0/11:0) for quantification. For quantification of lipids classes in CSF fractions, known amounts of lipids with a fixed 5 ng IS were run separately through the LC-MS/MS. Standard curves of the lipid amounts versus lipid/PC (11:0/11:0) intensities were acquired and used to quantify the lipids in the SF and NP fractions (ng/mL CSF) [53].

### 2.8. Statistical Methods

All analyses were performed using GraphPad Prism software (Version 7, GraphPad Software, Inc., La Jolla, CA, USA) and 0.05 significance level. All lipid data are presented as mean ± SEM (ng/mL). Student paired *t*-tests were used to determine differences between Aβ, Tau, lipids, and proteins among the clinical groups. We used Fisher’s exact tests for categorical variables describing the presence or absence of risk factors in our study groups. We used Spearman ranked analyses to assess the correlation between GP and SP classes with Aβ_42_, Tau, PLA_2_, nSMase, or aSMase. 

## 3. Results

### 3.1. Demographic Data and CSF Biomarkers

A total of 39 participants consented and were classified as having dementia. After clinical assessment, some participants met the criteria of LOAD (*n* = 29), while the rest were classified as other dementias (*n* = 10). The OD category was composed of DLB (*n* = 3), behavior variant FTD (*n* = 3), and vascular or mixed dementia (*n* = 4). Analyses of education and ApoE genotype numerical scale showed no group differences. Similarly, there was no significant difference in age, sex, body mass index (BMI), and neuropsychological classification between LOAD and OD (Table 1). Likewise, Aβ_42_ and Tau levels in the CSF were similar in LOAD compared with OD (Table 1). 

Typical for dementias, both groups had reduced Aβ_42_ and elevated Tau levels in contrast to age-match cognitively normal individuals (722 ± 299, 273 ± 149 pg/mL, respectively) [51]. Dementia duration was 39% less in OD compared with LOAD, suggesting that OD participants (Table 1) have a higher rate of disease progression.

### 3.2. Dementia Risk Factors and Medication Use

We examined the frequencies of some AD risk factors in LOAD and OD to ascertain that these did not contribute to any differences in lipid metabolism. Our data show that the occurrence of the most common risk factors was similar in the LOAD and OD populations (Table 2).

We examined the number of prescription and over the counter medications in our study populations. The mean number of prescription medications in LOAD (mean ± SD, 4.2 ± 2.3) did not significantly (*p* > 0.4) differ from that of OD (mean ± SD, 3.8 ± 3.5). Similarly, the number of over the counter supplements were similar (*p* > 0.6), suggesting that differences in medication usage might not account for metabolic differences in LOAD compared with OD.

### 3.3. GPs in LOAD and OD

To determine if lipid metabolism is different in CSF from LOAD compared with OD, we measured the GP contents of the SF and NP fractions using LC-MS/MS. Of several GP classes in SF, only phosphatidylserine (PS) levels were significantly (*p* < 0.05) higher in OD compared with LOAD (Table 3). In general, there is a decrease in PC and PE levels in SF and an increase in LPC. Although not significantly different, PC and PE levels were 54 % and 20 % higher in NP from OD compared with LOAD (Table 3).

### 3.4. Sphingolipids in LOAD and OD

We compared levels of SP classes in different CSF fractions to determine if SP metabolism is altered in LOAD compared to OD. CM levels in NP was higher for OD compared with LOAD, while levels of other SP classes were similar in both clinical groups, although we noticed a tendency for higher levels in OD compared with LOAD (Table 4).

### 3.5. Enzyme Activities of LOAD and OD CSF Samples

To determine mechanisms that may account for significant changes in lipids, we analyzed the activities of three enzymes using fluorescent assays. PLA_2_ and nSMase activity assays revealed no differences between LOAD and OD (Table 5).

However, we observed significantly higher aSMase activity in CSF from OD participants compared with LOAD (*p* < 0.05). The ratio aSMase/nSMase was higher in OD compared to LOAD (*p* < 0.02). 

### 3.6. Correlation of Glycerophospholipids and Aβ_42_ or Tau

To determine any link between lipid metabolism and AD pathology, we performed Spearman correlation analyses between GP classes and Aβ_42_ and Tau levels in CSF. We observed a negative relationship between the Aβ_42_ and LPC/PC in SF; Spearman ρ = −0.46 with *p* < 0.02 for LOAD samples. In addition, total Tau correlated with LPC in NP for LOAD participants (ρ = 0.45, *p* < 0.02). In contrast, only LPC in SF positively correlated with Tau in OD (ρ = 0.85, *p* < 0.01). 

### 3.7. Correlation of Sphingolipids and Aβ_42_ or Tau

There were no statistically significant correlations noted between Aβ_42_ or Tau with SF or NP sphingolipids from LOAD study participants. In contrast to LOAD, Tau negatively correlated with CM in the SF fraction (ρ = −0.75, *p* < 0.03) in OD. Also, Tau levels negatively correlated to SM levels in supernatant fluids (ρ = −0.77, *p* < 0.03).

### 3.8. Correlation of Enzyme Activities with CSF Lipids

**Figure 1:**
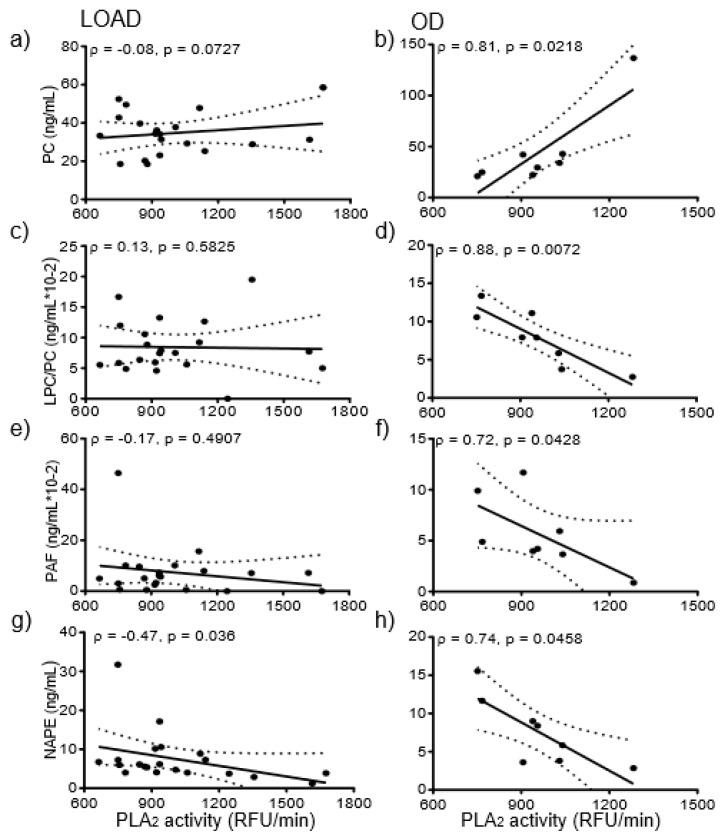
Correlations between PLA_2_ activity and GPs- LOAD demonstrated no significant correlation between PLA_2_ activity and PC in NP (**a**). However, PLA_2_ positively correlated with PC in NP in OD samples (ρ = 0.81, *p* < 0.03) (**b**). PLA_2_ activity in CSF from LOAD subjects did not correlate with NP LPC/PC (**c**), while PLA_2_ activity of OD negatively correlated (ρ = −0.88, *p* < 0.01) with NP LPC/PC (**d**). LOAD PLA_2_ activity did not correlate with NP PAF (**e**), while OD PLA_2_ activity negatively correlated (ρ = −0.74, *p* < 0.05) with NP PAF (**f**). PLA_2_ activity negatively correlated with NAPE in both LOAD (ρ = −0.47, *p* < 0.05) (**g**) and OD (ρ = −0.47, *p* < 0.05) (**h**). PLA_2_ activity was available for eight OD because of CSF limitation for two OD samples.

We next determined whether enhanced lipase activity accounted for metabolic differences between LOAD and OD. PLA_2_ activity correlated with several SF and NP lipids in CSF from OD participants. For OD samples, LPC in SF positively correlated with PLA_2_ activity in CSF (ρ = 0.81, *p* < 0.03), but no such correlation was evident for LOAD. Similarly, xPE levels in SF correlated with PLA_2_ activity (ρ = 0.86, *p* < 0.02) in OD but not in LOAD. PLA_2_ activity in LOAD did not correlate with NP PC (Figure 1a), while PLA_2_ activity positively correlated with NP PC in OD (Figure 1b). 

Similarly, PLA_2_ activity did not correlate with LPC/PC in LOAD (Figure 1c) but negatively correlated (ρ = −0.88 (*p* < 0.01) with LPC/PC of OD (Figure 1d). Finally, PLA_2_ activity in LOAD did not correlate with platelet-activating factor-like lipids (PAF-LL) (Figure 1e) but negatively correlated with NP PAF-LL in OD (Figure 1f). The only correlation that was significant across both LOAD and OD was between PLA_2_ activity and NAPE and only in the NP fractions. PLA_2_ activity in LOAD negatively correlated with NAPE in NP (ρ = −0.47, *p* < 0.04) (Figure 1g). NP NAPE levels negatively correlated (ρ = −0.74, *p* < 0.05) with the activity of PLA_2_ (Figure 1h). 

We also determined the correlation of SP levels in CSF fractions from OD participants with aSMase or nSMase activities. Aβ_42_/Tau ratio was significantly related to nSMase activity (ρ = 0.68, *p* < 0.04). NP-SM was negatively related to the activity of aSMase (ρ = −0.68, *p* < 0.05), and NP dhCM was positively correlated with the activity of nSMase (ρ = 0.70, *p* < 0.03).

## 4. Discussion

Our objective is to determine if there are any differences in lipid metabolic pathways in CSF from subjects with LOAD compared with those with OD. In this study, the OD population is made up of participants with DLB, FTD, VaD, and mixed dementia that can often be clinically differentiated from LOAD by the earlier age of onset, step-wise progression, and shorter survival time [48]. OD subjects have a median survival time from symptom onset of 6 ± 1.1 years compared with LOAD subjects who have a survival time from presentation of 11.7 years [56]. In our age-matched study population, duration of dementia is shorter in OD than in LOAD (Table 1). Given the accelerated rate of progression and death in OD, the degree of lipid metabolic abnormality may be different between OD and LOAD. Our study confirms this by demonstrating changes in GP, SP, and enzymes that metabolize important inflammatory and apoptotic lipids. 

Examining GP classes, the only significant variation was PS that almost tripled in the SF of OD compared with LOAD. Typically found in the inner leaflet of plasma membranes and mitochondrial-associated membranes, PS only constitutes 2–10% of total lipids [57,58]. Although present in small amounts, PS is crucial in the signaling apoptosis [59]; cells externalize PS to the outer leaflet as a recognition signal to phagocytic cells or to initiate the blood clotting cascade [60]. The increase in PS in SF may indicate accelerated neurodegeneration in OD compared with LOAD study participants: OD brain cells exposed to higher PS levels are undergoing apoptosis faster than LOAD. Higher PS may account for the earlier clinical manifestation and faster cognitive decline in participants with OD. 

Our data also indicate that there is a disruption in GP metabolism in LOAD and OD that may impact Aβ_42_ and Tau metabolism. In regards to the correlation in LOAD between Aβ_42_ and LPC/PC in SF, alteration in lipid metabolism may influence the proper clearance of Aβ_42_, resulting in the pathological features associated with Aβ_42_ aggregation [61]. The indication, then, is that those with higher levels of Aβ_42_ in CSF have a lower disturbance of lipid metabolism. In contrast, highly perturbed lipid membranes do not only result in abnormal clearance of Aβ_42_ but increase the susceptibility of amyloid precursor protein (APP) to digestion by secretases [62,63]. We also found that higher levels of Tau, that indicate pathology found in dementia, positively correlated with LPC in both LOAD and OD CSF samples. Since LPC is derived from PC by PLA_2_-catalyzed lipolysis, induces myelination, and disrupts the blood-brain barrier [64], these data suggest that disruption of membrane structure is associated with dementia. Our data are in agreement with several studies showing that many PLA_2_ isoforms contribute to AD pathology [23,65,66,67]. 

SPs are another group of lipids essential in brain function and implicated in AD pathology [53,68]. Of the major SP classes detected in CSF, only the levels of CM was higher in NP of OD compared with LOAD. As a critical component for the initiation of apoptosis and autophagy, CM acts on several cells, including neurons [31,69,70]. Thus, higher levels of CM can cause increased rates of apoptosis in neural cells in OD compared with LOAD. This faster cell death would cause the clinical presentation to be identified earlier in OD compared to LOAD participants. 

Further evidence for a role of SPs in OD is shown when Tau levels negatively correlate with CM and SM in SF. How Tau interacts with lipids has not been widely studied, but one can conclude that SP metabolism associates with Tau and this interaction is different in LOAD compared with OD. 

Several enzymes modify GPs and SPs to form lipid mediators of inflammation or lipids that modify neuronal function. PLA_2_ is notable for its role in Alzheimer’s disease and other neurological disorders, due to the release of the inflammatory precursor arachidonic acid [67,71]. Once released, cyclooxygenase, lipoxygenase, and cytochrome p450 convert arachidonic acid to bioactive lipid mediators of inflammation. These PLA_2_- and COX-derived products are crucial in regulating an inflammatory response in LOAD [72]. Inflammatory cytokines can induce the synthesis of PLA_2_ and also stimulate the de novo synthesis of ceramide and PAF [73,74,75,76]. In our study, PLA_2_ activity in CSF from OD participants correlated with levels of several GPs; LPC and xPE in SF, and PC, PAF, NAPE, and LPC/PC in the NP fraction. PAF is an inflammatory GP produced and metabolized through two variations of PLA_2_—cytosolic and Lp-PLA_2_ [77]. LPC interacts with inflammatory cells to increase cytokine production, such as tumor necrosis factor alpha (TNF-α) and interleukin (IL)-6 [78]. LPC appears to be a direct reflection on the intensity of reactive oxygen species present at the inflammatory site and can induce localized demyelination in rats [77,79]. A higher ratio of LPC/PC indicates that greater inflammatory markers are present in the CSF of OD patients. Correlation of PLA_2_ with these lipids suggests that hydrolysis may be linked with destructive neuro-inflammation in subjects with OD. 

NAPE is a significant component of intracellular membranes and has been shown to accumulate under pathological conditions that involve the degradation of membranes [80,81]. NAPE maintains proper physiological membrane configurations [81]. As NAPE accumulates in the brain tissue of LOAD and OD, fewer NAPE molecules will be removed in the CSF. The negative correlation between NAPE and PLA_2_ observed in LOAD and OD reflects a disturbance in neuronal membrane lipids. 

The activities of aSMAse and aSMase/nSMase ratio were higher in CSF from subjects with OD compared to LOAD. aSMase and nSMase are both involved in the degradation of SM to form CM, a known pro-apoptotic biolipid [31,82,83]. Functional consequences of SM degradation include apoptosis, growth arrest, and decreased protein secretion [84]. OD also demonstrated a significant negative correlation between aSMase activity and NP SM. The higher aSMase activity in OD compared with LOAD may account for elevated CM levels. Thus, higher aSMase activity in OD can result in faster apoptosis than in LOAD. A second positive correlation is noted between nSMase and dhCM. dhCM is a precursor to CM, and increased levels of dhCM are associated with oxidative stress [34,85]. Therefore, oxidative stress linked with lipid metabolic dysregulation may associate with AD pathophysiology. 

## 5. Conclusions

This pilot cross-sectional study shows that lipid metabolism in CSF fractions differs between LOAD and OD. The small OD sample size limits our ability to examine previously described lipidomic variations between mixed dementia phenotypes [86]. These studies of lipid metabolism can provide insight into the dysfunction of metabolic pathways that can be used for diagnosis or to target AD therapy [87,88]. Replication with bigger sample size and consideration of different age of onset for defined dementia variants will be needed to associate known clinical phenotypes with specific lipid metabolic changes in CSF fractions. 

## Figures and Tables

**Table 1:** Demographics and CSF biomarkers of LOAD and OD.

Categories	LOAD	OD	^1^ Difference (% LOAD)
Number of Subjects	29	10	
Female (%)	53%	40%	
	Mean ± SEM		
Age (years)	77.4 ± 1.8	77.3 ± 4.0	−0.1
Education (years)	14.5 ± 2.8	14.0 ± 3.1	−3.5
ApoE Genotype	3.6 ± 0.2	4 ± 0	11.4
BMI	1.6 ± 0.2	1.3 ± 0.3	−19.4
^2^ Neuropsych Classification	9.1 ± 0.2	8.8 ± 0.3	−3.72
MMSE	15 ± 1.5	16.0 ± 2.7	7.5
CSF Tau (pg/mL)	472.6 ± 41.1	387.2 ± 100.6	−18.1
CSF Aβ_42_ (pg/mL)	506.2 ± 43.1	432.3 ± 59.4	−13.8
Aβ_42_/Tau	1.4 ± 0.2	1.2 ± 0.3	−12.1
Dementia duration (years)	5.7 ± 0.9	3.5 ± 0.6	−39.0

^1^ Difference of means = (OD–LOAD)/LOAD × 100. ^2^ Neuropsychology scale > 8 indicates dementia. LOAD: Late-onset Alzheimer’s disease; OD: Other Dementia; BMI: body mass index; MMSE: Mini-Mental State Examination; CSF: cerebrospinal fluid.

**Table 2:** Risk factors in late-onset Alzheimer’s disease (LOAD) and other dementia (OD) participants.

Risk Factors	LOAD *n* (Frequency), %	OD *n* (Frequency), %	*p* Value ^1^
Obesity ^#^	26 (3), 11.5	9 (0), 0	0.55
Hyperlipidemia	28 (9), 32.1	10 (4), 40	0.71
Hypertension	27 (15), 55.6	10 (4), 40	0.48
Heart disease	28 (11), 39.3	10 (4), 40	0.99
Diabetes	29 (4), 13.9	10 (1) 10	0.99
Family history of dementia	29 (14), 48.3	10 (2), 20	0.15

^1^ Fisher’s exact test. ^#^ BMI > 30.

**Table 3:** Glycerophospholipids (GPs) in supernatant fluid (SF) and nanoparticle (NP).

**GPs in SF (ng/mL)**	**LOAD**	**OD**	**^1^ Difference (% LOAD)**
LPC	15.6 ± 1.1	16.0 ± 2.1	2.8
PC	8169.1 ± 915.1	7064.3 ± 1238.3	−13.5
LPC/PC	0.0022 ± 0.0002	0.0023 ± 0.0002	4.6
PS	6.2 ± 1.0	16.01 ± 6.6	149.4 *
NAPE	10.6 ± 2.3	15.09 ± 3.1	42.6
PE	27.8 ± 3.2	16.4 ± 2.5	−41.2
xPE	1.3 ± 0.1	1.40± 0.2	11.2
**GPs in NP (ng/mL)**	**LOAD**	**OD**	**% LOAD**
LPC	3.05 ± 0.3	2.7 ± 0.2	−11.2
PC	40.05 ± 4.8	61.9 ± 23.4	54.5
LPC/PC	0.09 ± 0.01	0.08 ± 0.01	−10.1
PAF	0.08 ± 0.02	0.06 ± 0.01	−28.3
NAPE	6.8 ± 1.1	6.9 ± 1.3	0.8
PE	7.9 ± 0.6	9.5 ± 1.3	20.5
xPE	1.8 ± 0.5	1.7 ± 0.2	−7.3

The data are the mean ± SEM, ng/mL. The *p* values compare LOAD to OD and * denotes *p* < 0.05. ^1^ Difference of means as a percentage of LOAD = (OD–LOAD)/LOAD × 100. LPC: lysophosphatidylcholine; PC: phosphatidylcholine; LPC/PC: lysophosphatidylcholine to phosphatidylcholine ratio; PS: phosphatidylserine; NAPE: N-acyl phosphatidylethanolamine; PE: phosphatidylethanolamine; xPE: unknown lipid containing phosphoethanolamine; PAF, platelet-activating factor.

**Table 4:** Sphingolipids (SP) in supernatant fluid (SF) and nanoparticle (NP) of late-onset Alzheimer’s disease (LOAD) and other dementia (OD).

**SP Classes in SF**	**LOAD (ng/mL)**	**OD (ng/mL)**	**^1^ Difference (% LOAD)**
SM	1888.2 ± 138.2	1892.1 ± 400.1	2.1
CM	60.0 ± 6.9	73.2 ± 20.9	22.2
dhCM	179.2 ± 18.9	186.3 ± 63.7	4.0
CM/SM	0.0309 ± 0.0022	0.0369 ± 0.0041	19.4
**SP Classes in NP**	**LOAD (ng/mL)**	**OD (ng/mL)**	**% LOAD**
SM	95.2 ± 8.1	110.7 ± 20.6	16.3
CM	5.9 ± 0.6	6.5 ± 0.4	9.4 *
dhCM	53.9 ± 3.0	55.6 ± 5.7	3.2
CM/SM	0.07 ± 0.01	0.08 ± 0.01	2.7

These data are the mean ± SEM, ng/mL. The *p* values compare sphingolipids in LOAD versus OD and * denotes *p* < 0.05. ^1^ Difference of means as the percentage of LOAD = (OD–LOAD)/LOAD × 100. SM: sphingomyelin; CM: ceramide; dhCM: dihydroceramide; CM/SM: ceramide to sphingomyelin ratio.

**Table 5:** Phospholipase A2 (PLA_2_), neutral sphingomyelinase (nSMase), and acid sphingomyelinase (aSMase) activities in the cerebrospinal fluid (CSF) of late-onset Alzheimer’s disease (LOAD) and other dementia (OD) groups.

Enzymes	LOAD (RFU/min)	OD (RFU/min)	^1^ Difference (% LOAD)
PLA_2_	1008.1 ± 59.2	959.2 ± 60.1	−4.9
nSMase	24.3 ± 2.2	20.4 ± 1.9	−18.9
aSMase	15.0 ± 1.5	20.7 ± 1.7	37.9 *
aSMase/nSMase	0.8 ± 0.7	1.1 ± 0.1	39.3 *

^1^ Difference of means as a percentage of LOAD = (OD–LOAD)/LOAD × 100 and * denotes *p* < 0.05.

## Abbreviations

CSF	cerebrospinal fluid
CM	ceramide
dhCM	dihydroceramide
LC	liquid chromatography
LOAD	late-onset Alzheimer’s Disease
LPC	lysophosphatidylcholine
PAF_LL	platelet-activating factor-like lipids
PE	phosphatidylethanolamine
PC	phosphatidylcholine
PS	phosphatidylserine
GP	glycerophospholipid
PLA_2_	phospholipase A_2_
NAPE	N-acyl phosphatidylethanolamine
NP	nanoparticles
SM	sphingomyelin
SMase	sphingomyelinase
SP	sphingolipids
SF	supernatant fluid
xPE	unknown lipid containing phosphoethanolamine
